# Paroxysmal Sneezing and Hay Asthma

**Published:** 1909-07-10

**Authors:** 


### Diseases of Children.

### PAROXYSMAL SNEEZING AND HAY ASTHMA.

The occurrence of paroxysmal sneezing in children is not very common. A typical instance was that of a little girl, three years old, who had been subject to attacks since the end of the first year of life. Previous to that she had suffered with facial eczema. The paroxysms would come on in the mornings, on change of temperature, on driving, and at all times of the year. The mother regarded the smell of horses as a definitely exciting cause. From 15 to 20 sneezes were given in rapid succession; the child yawned frequently, and seemed dull and irritable afterwards. A spray of adrenalin solution, 1 in 1,000, was beneficial, but not curative. Subsequently some adenoids and portions of the inferior turbinal bones were removed. At the operation it was found that irritation of the mucosa over these bones induced sneezing; but a month later the attacks were as bad as ever. There was a strong family history of hay fever. A younger child subsequently passed through a severe attack of general eczema, lasting from three to eight months of age, and a month later had true spasmodic asthma. In this case there is no doubt that the affection belongs to the group of asthmatic conditions.

Paroxysmal sneezing is sometimes spoken of as paroxysmal coryza, vaso-motor coryza, or vaso-motor rhinitis. It is essentially the same as hay fever, and due to irritation of the terminal filaments of the nasal nerve; occasionally to the irritation of a spur on a turbinal. Patches of hyperæsthesia of the nasal mucosa may be found on examination, just as in true asthma. A neurotic or gouty ancestry, sometimes combined, is the common hereditary predisposing factor. Exciting causes include innumerable aerial emanations, varying in their effect in different individuals, east winds, and gastro-intestinal troubles, as well as local nasal affections.

Hay fever is sometimes regarded as dependent on an abnormally large antrum of Highmore. Another view of its pathology is that it is primarily conjunctival. Many attacks begin with itchiness of each inner canthus and irritation in the nose. Examination reveals slight hyperæmia of the bulbar conjunctiva, a fan-shaped arrangement of vessels, analogous to pterygium, which is merely an exaggerated condition of the same affection. The eyelid is red and swollen, and lachrymation and photophobia are present. It is quickly followed by sneezing, nasal obstruction, and profuse watery nasal secretion. The whole duration of the attack is about three weeks. Thus it differs from paroxysmal sneezing in its extent, the ocular involvement, and its duration. No doubt there are varieties of these affections, and one passes through intermediate gradations of severity into the other. At one end of the scale is the simple paroxysm of sneezing, such as is induced in normal persons by nasal irritation, and at the other the severe recurrent attacks of hay fever. Possibly in the one instance the mucous membrane of the nose is alone susceptible to the irritant, while in the other the conjunctival is equally, if not more, susceptible.

Treatment necessarily varies somewhat in accordance with the variety of the affection with which we have to deal. If the case is merely one of paroxysmal sneezing, it is obviously advisable to remedy any local condition of the nose or throat, which may possibly act as an exciting cause or interfere with proper breathing. Similarly, it is advisable to use such remedies as will render the nasal mucosa less sensitive to irritation or vaso-motor turgescence. Unfortunately the effects of treatment on these lines passes off quickly. A spray of adrenalin solution is undoubtedly the best immediate remedy. Any local tender or hyperæsthetic spot on the nasal mucosa can be painted with silver nitrate solution, 2 per cent. strength, or touched with the galvano-cautery. Adenoids and enlarged tonsils should be removed and proper respiratory exercises insisted upon. Considerable benefit is also derived from definite training in the exposure to sudden changes in temperature. It is doubtful, however, whether any advantage will be derived by attempting to train the patient to bear exposure to the particular exciting aerial emanation which brings on the paroxysm.

On the asumption that some cases of hay fever are primarily conjunctival in origin, and in the presence of evidence of such irritation at the onset of an attack, the eyes should be protected by means of glasses from exposure to dust. Dunbar's anti-toxin or serum, "pollantin," is instilled in fluid form into the eyes, and as a powder into the nose. Apparently it is occasionally useful. Cocaine sprays ought rarely to be employed, because of the danger of setting up the cocaine habit. The drug sets up local constriction, but patients, who are old enough, get into the habit of inhaling the spray at intervals all day long. The drug is found in some of the advertised asthma cures. A lotion of resorcin grs. ii. to iv., sodium chloride grs. iv., in an ounce of water is a valuable nasal astringent. It may be more efficacious if a couple of minims of acetic acid are added to it. Internal treatment is absolutely valueless in simple paroxysmal sneezing, and not of much use in hay faver. The drugs chiefly recommended are quinine, extract of belladonna, extract of stramonium, and arsenic. It is of importance to attend to the general health and the alimentary tract.

Climatic treatment is of value through avoidance of the particular irritant—*e.g.* high altitudes and sea voyages, if the cause is the pollen of certain grasses. An equable temperature is advantageous when attacks are induced by sudden changes in the temperature of the atmospheric air alone. For intractable cases the resection of the nasal nerve has been suggested. This nerve is widely distributed. It supplies the conjunctiva, integument of the eyelids, lachrymal caruncle and sac, ciliary muscle, cornea, frontal sinus, and the mucosa of the anterior portions of the nose. All the symptoms of severe hay fever are explicable in accordance with the distribution of this nerve, and ought to subside if it is resected. The operation would have to be bilateral, for it is rare for the affection to be unilateral.

